# A decision tree algorithm to identify predictors of post-stroke complex regional pain syndrome

**DOI:** 10.1038/s41598-024-60597-3

**Published:** 2024-04-30

**Authors:** Yuichi Katsura, Satoshi Ohga, Kazuhiro Shimo, Takafumi Hattori, Tsukasa Yamada, Takako Matsubara

**Affiliations:** 1 Faculty of Rehabilitation, Kobe Gakuin University Graduate School, 518, Arise, Ikawadani-cho, Nishi-ku, Kobe, Hyogo 651-2180 Japan; 2 Department of Rehabilitation, Kishiwada Rehabilitation Hospital, 8-10, Kanmatsu-cho, Kishiwada-shi, Osaka, 596-0827 Japan; 3 Faculty of Rehabilitation, Kobe Gakuin University, 518, Arise, Ikawadani-cho, Nishi-ku, Kobe, Hyogo 651-2180 Japan

**Keywords:** Medical research, Signs and symptoms

## Abstract

This prospective cohort study aimed to identify the risk factors for post-stroke complex regional pain syndrome (CRPS) using a decision tree algorithm while comprehensively assessing upper limb and lower limb disuse and physical inactivity. Upper limb disuse (Fugl-Meyer assessment of upper extremity [FMA-UE], Action Research Arm Test, Motor Activity Log), lower limb disuse (Fugl-Meyer Assessment of lower extremity [FMA-LE]), balance performance (Berg balance scale), and physical inactivity time (International Physical Activity Questionnaire-Short Form [IPAQ-SF]) of 195 stroke patients who visited the Kishiwada Rehabilitation Hospital were assessed at admission. The incidence of post-stroke CRPS was 15.4% in all stroke patients 3 months after admission. The IPAQ, FMA-UE, and FMA-LE were extracted as risk factors for post-stroke CRPS. According to the decision tree algorithm, the incidence of post-stroke CRPS was 1.5% in patients with a short physical inactivity time (IPAQ-SF < 635), while it increased to 84.6% in patients with a long inactivity time (IPAQ-SF ≥ 635) and severe disuse of upper and lower limbs (FMA-UE score < 19.5; FMA-LE score < 16.5). The incidence of post-stroke CRPS may increase with lower-limb disuse and physical inactivity, in addition to upper-limb disuse. Increasing physical activity and addressing lower- and upper-limb motor paralysis may reduce post-stroke CRPS.

## Introduction

Complex regional pain syndrome (CRPS) is a neuropathic pain disorder caused by painful trauma, nerve lesions, immobilization of the extremities, stroke, spinal cord injury, and myocardial infarction^1,2^. CRPS is classified based on the absence (type I) or presence (type II) of peripheral nerve injury^3^. Post-stroke CRPS, formerly referred to as shoulder-hand syndrome, is classified as type I due to the lack of obvious peripheral nerve injury^4^. Post-stroke CRPS presents with various symptoms such as pain, hyperalgesia, allodynia, swelling, a limited range of motion of the wrist and hand, edema, and warmth and redness of the wrist and hand^4^. Previous studies have reported that the prevalence of post-stroke CRPS ranges from 12.5 to 50.0%^5–8^. Its prevalence within 6 months after stroke onset is approximately 30% and it is one of the most common complications. Currently, there are no guidelines for the prevention of post-stroke CRPS. Therefore, early diagnosis and treatment of post-stroke CRPS are essential for pain reduction, leading to functional improvement after stroke. However, the pathophysiological mechanism of post-stroke CRPS remains unclear.

Several previous studies have reported shoulder subluxation and severe upper-limb motor impairment as risk factors for developing post-stroke CRPS^6,9,10^. Furthermore, we previously showed that inactivity of the affected upper limb influenced the development of post-stroke CRPS^8^. Limb immobilization is considered a risk factor for developing CRPS type I, even among healthy animals and humans^11,12^. Therefore, disuse due to shoulder subluxation and severe upper-limb motor impairment may also be risk factors for post-stroke CRPS. However, patients with upper limb disuse following shoulder subluxation or severe motor paralysis do not necessarily develop post-stroke CRPS in clinical situations. A previous study reported that prolonged periods of physical inactivity, including in the non-affected area, exacerbated pain and disability in patients with pain^13^. Post-stroke motor paralysis often occurs in the upper and lower limbs, leading to systemic physical inactivity, which may be considered a risk factor for post-stroke CRPS. However, no study has examined whether these factors are involved in the development of post-stroke CRPS.

The application of artificial intelligence, referred to as machine learning (ML), has expanded to include stroke treatment^14^. Previous research applied ML to identify variables that predict treatment responsiveness in patients with knee osteoarthritis^15^. Among these ML algorithms, the decision tree algorithm has been attractive for clinical application because of its transparency and high visibility^16^. The decision tree algorithm is a data-mining technique that can be applied irrespective of the scale of explanatory variables. It subdivides successive cases into independent groups based on the values of the independent variables, ultimately categorizing them into distinct groups. The tree diagram is organized in a hierarchical manner, with the most significant relationships at the top, to facilitate understanding of the interrelationships between factors. Applying this to identify patients with a high probability of developing post-stroke CRPS may be useful for rehabilitation programs to prevent the development of post-stroke CRPS.

This study aimed to identify risk factors, such as disuse of the lower limb due to motor paralysis and physical inactivity in addition to upper limb disuse, which influence the development of post-stroke CRPS using the ML method.

## Results

**Figure 1:**
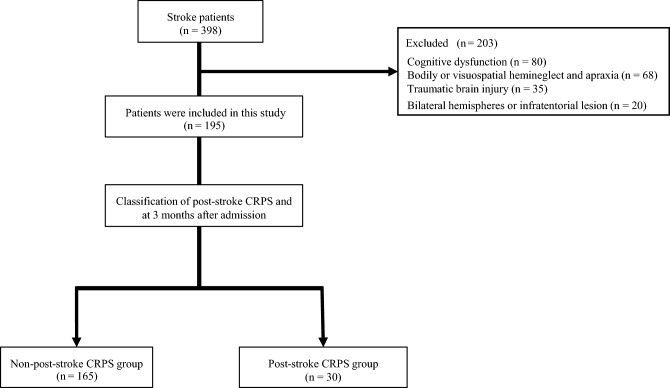
Flow chart of the study participants. CRPS, complex regional pain syndrome.

During the study period, 398 patients with stroke were admitted to our hospital, among whom 203 patients were excluded. The reasons for exclusion were cognitive dysfunction (n = 80), bodily or visuospatial hemineglect and apraxia (n = 68), traumatic brain injury (n = 35), and bilateral hemispheres or infratentorial lesions (n = 20). Thus, 195 patients were included in this study. Overall, 30 and 165 patients were assigned to the post-stroke CRPS and non-post-stroke CRPS groups, respectively (Fig. 1). The incidence of post-stroke CRPS 3 months after admission was 15.4%.

### Demographic and clinical data

The demographic and clinical data at admission are presented in Table 1. No significant differences were observed between the non-post-stroke CRPS and post-stroke CRPS groups in terms of age, sex, stroke etiology, lesion side, days since stroke onset, and Mini-Mental State Examination (MMSE) scores. However, the post-stroke CRPS group exhibited lower scores in Fugl-Meyer assessment of upper extremity (FMA-UE), Fugl-Meyer Assessment of lower extremity (FMA-LE), Action Research Arm Test (ARAT), Motor Activity Log (MAL) at amount of use scale (AOU) and quality of movement scale (QOM), and Berg balance scale (BBS) compared to the non-post-stroke CRPS group (all p < 0.01). Additionally, International Physical Activity Questionnaire-Short Form (IPAQ-SF) at sitting and lying position times were higher in the post-stroke CRPS group (p < 0.01). Pain intensity in the post-stroke CRPS group was 5.6 ± 1.3 points, as assessed by the numerical rating scale (NRS).

**Table 1:** Demographic and clinical data of each group at the admission.

Variables	Non-post CRPS group (n = 165)	Post-stroke CRPS group (n = 30)	*P-value*
Age	71.3 ± 10.2	71.9 ± 8.8	0.725
Sex, male (%)	77 (47.0)	16 (53.3)	0.554
Etiology of stroke			0.692
Infraction (%)	84 (51.0)	17 (56.7)	
Hemorrhage (%)	81 (49.0)	13 (43.3)	
Side of lesion, right (%)	78 (47.3)	12 (40.0)	0.552
Time since stroke, days	32.3 ± 10.1	30.6 ± 8.4	0.377
MMSE (30)	26.7 ± 2.3	26.2 ± 1.6	0.344
Fugl-Meyer assessment			
Upper extremity (66)	40.0 ± 24.3	8.4 ± 5.6	< 0.001*
Lower extremity (34)	24.9 ± 7.4	11.9 ± 7.6	< 0.001*
ARAT (57)	32.8 ± 24.5	3.2 ± 4.2	< 0.001*
MAL			
AOU (5)	2.4 ± 2.1	0.1 ± 0.2	< 0.001*
QOM (5)	2.3 ± 2.2	0.0 ± 0.1	< 0.001*
BBS (56)	36.2 ± 11.1	19.3 ± 8.7	< 0.001*
IPAQ-SF (sitting and lying positions times; min/day)	544.5 ± 103.0	705.2 ± 56.4	< 0.001*
NRS (10)	–	5.6 ± 1.3	< 0.001*

NOTE. Scores are mean ± SD, or n (%). Significance at P < .05.

MMSE, Mini Mental State Examination; ARAT, Action Research Arm Test; MAL at AOU, Motor Activity Log at amount of use; MAL﻿ at QOM, motor activity log at quality of movement; BBS, Berg balance scale; IPAQ-SF, International Physical Activity Questionnaire-Short Form; NRS, numerical rating scale.

### Prediction of post-stroke CRPS development

**Figure 2:**
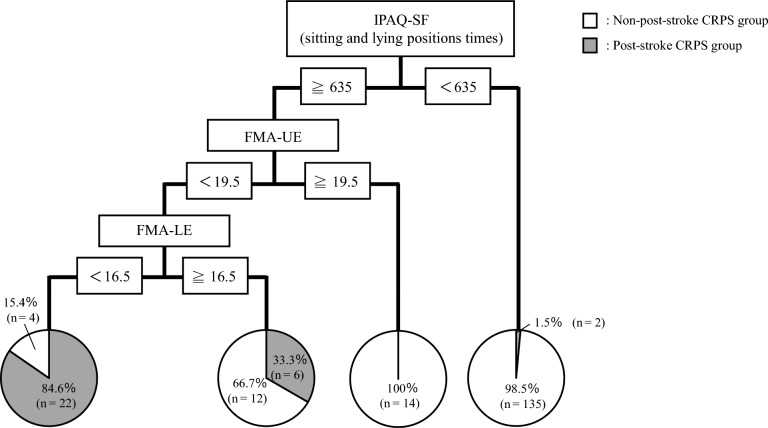
Prediction of post-stroke CRPS development. CRPS, complex regional pan syndrome; IPAQ-SF, International Physical Activity Questionnaire-Short Form; FMA-UE, Fugl-Meyer assessment of the upper extremity; FMA-LE, Fugl-Meyer assessment of the lower extremity.

The classification and regression tree (CART) model automatically identified IPAQ-SF scores at sitting and lying positions, FMA-UE, and FMA-LE as discriminators for the development of post-stroke CRPS (Fig. 2). The incidence rates were 1.5% for low physical inactivity, 0% for high inactivity with low severity of upper limb disuse, 33.3% for high inactivity with high severity of upper limb disuse and low severity of lower limb disuse, and 84.6% for high inactivity with high severity of both upper and lower limb disuse.

## Discussion

In this study, we developed a CART model using a decision tree algorithm to identify the risk factors for developing post-stroke CRPS in patients with stroke. The results of the CART analysis suggested that physical inactivity time and the severity of upper and lower limb disuse could discriminate the development of post-stroke CRPS. Previous studies have focused only on the affected upper limb function^17^. However, our results indicate that severe lower limb disuse and systemic physical inactivity, including in the non-affected area, are associated with the development of post-stroke CRPS. We found that patients who had upper-limb disuse and a combination of lower-limb disuse and systemic physical inactivity before developing CRPS were more likely to develop post-stroke CRPS. Moreover, the results of the decision tree analysis identified physical inactivity time (IPAQ-SF at sitting and lying positions ≥ 635 min/day) and upper and lower limb disuse (FMA-UE score < 19.5; FMA-LE score < 16.5) as risk factors for the development of post-stroke CRPS.

The incidence of post-stroke CRPS was very low (1.5%) when the patients had low physical inactivity before developing CRPS. A previous study reported that since systemic physical inactivity is a risk factor for the development of musculoskeletal pain, it is important to increase physical activity^18^. Our results suggest that systemic physical inactivity is also a risk factor for pain in patients with stroke, and increasing physical activity may prevent the development of post-stroke CRPS. Furthermore, patients with high physical inactivity without severe upper limb disuse before developing CRPS did not develop post-stroke CRPS. However, the incidence of post-stroke CRPS increased to approximately 30% among patients with high physical inactivity and severe upper limb disuse without severe lower limb disuse. The incidence of post-stroke CRPS increased significantly to approximately 80% with high physical inactivity and severe upper and lower limb disuse. Our results suggest that although systemic physical inactivity is a risk factor for the development of post-stroke CRPS, the coexistence of severe upper and/or lower limb disuse in the affected area greatly increases the incidence of post-stroke CRPS. Therefore, to prevent the development of post-stroke CRPS, it is necessary to assess and approach the affected upper limb function and the lower limb function without causing pain symptoms and systemic inactivity.

A recent meta-analysis reported that the development of post-stroke CRPS is associated with severe upper-limb motor paralysis, spasticity, and shoulder joint subluxation^17^. Particularly, the disuse of the paralyzed upper limb may be involved in the development of post-stroke CRPS. In this study, we also found that severe disuse of the upper limb was associated with the development of post-stroke CRPS. Previous studies on CRPS animal models have reported that limb immobilization causes peripheral changes in nerve fiber density, inflammatory mediator production, and neuropeptide signaling^19–21^. These changes lead to persistent nociceptive stimulation, leading to central changes, such as central sensitization in the spinal cord. Ultimately, these peripheral and central changes result in an increased reactivity to nociceptive stimuli, including allodynia and hyperalgesia. These peripheral and central changes due to the disuse of the paralyzed upper limb may also be involved in the mechanisms underlying the development of post-stroke CRPS.

Previous studies have indicated that some patients with upper limb inactivity do not develop post-stroke CRPS^5,8^, suggesting that additional risk factors may contribute to the development of post-stroke CRPS beyond upper limb inactivity. In this study, we found that lower-limb disuse not causing pain symptoms and systemic physical inactivity are also risk factors for the development of post-stroke CRPS. A previous meta-analysis examining the factors related to physical activity in patients with stroke showed that physical function, including lower limb function, influences the amount of physical activity^22^. In patients with stroke, systemic physical inactivity during hospitalization is often observed and affects the functional outcomes^23,24^. Additionally, systemic physical inactivity has been reported as a risk factor for the development of chronic pain^18^. No report has shown that physical activity is associated with the development of pain in patients with stroke; however, this study has shown such a relationship. In the state of systemic physical inactivity, M1 macrophages, which release inflammatory cytokines that activate nociceptors, increase, whereas M2 macrophages release anti-inflammatory cytokines that suppress nociceptors in the immune system in the skeletal muscle^18^. Additionally, previous studies have reported the association between physical inactivity and decreased brain activity in the dorsolateral prefrontal cortex, thalamus, and superior frontal gyrus^25^. These regions are involved in pain regulation, indicating that physical inactivity could lead to pain inhibitory dysfunction^26^. In fact, regular physical activity reduces pain intensity and the mechanisms involved in the anti-inflammatory immune and central nervous systems through the activation of endogenous opioids and cannabinoids; nevertheless, the central nervous system is impaired in patients with chronic pain and physical inactivity^18,27^. Thus, the inflammatory changes extending from peripheral tissues to the entire body, along with pain inhibitory dysfunction resulting from systemic physical inactivity, may represent a potential mechanism behind the development of post-stroke CRPS. This study indicated that the risk of developing post-stroke CRPS is increased in patients with a combination of inactivity in the affected upper and lower limbs and systemic physical inactivity, suggesting that peripheral and central sensitization in the affected area and the immune and central nervous systems in the systemic area may be involved in the mechanism of developing post-stroke CRPS.

The present study has certain limitations. First, the results were obtained from a single hospital. Multicenter studies are required to confirm the general applicability of our results. Second, we analyzed a small number of patients with post-stroke CRPS; therefore, we could not perform a multivariate analysis to exclude the influence of confounding factors. Lastly, neuroanatomical investigations of the brain were not performed. Previous studies have reported an association between post-stroke CRPS and the corticospinal tract, and the brain injury area may have affected our results^9^. Despite these limitations, this was the first study to simultaneously use various risk factors for the development of post-stroke CRPS, such as upper and lower limb disuse and systemic physical inactivity, and analyze them using a decision tree algorithm.

In conclusion, this study identified the severity of upper- and lower-limb disuse and systemic physical inactivity, including the non-affected area, as risk factors for the development of post-stroke CRPS. Furthermore, the combination of upper- and lower-limb disuse and systemic physical inactivity significantly increased the risk of developing post-stroke CRPS. Therefore, increasing physical activity and addressing lower- and upper-limb motor paralysis may reduce post-stroke CRPS.

## Methods

### Patients

This prospective cohort study included 195 patients with first-ever stroke. The patients who visited the Kishiwada Rehabilitation Hospital in Osaka, Japan, between June 2019 and October 2021 were enrolled in this study. The physician diagnosed stroke via neurological examination, brain computed tomography, or magnetic resonance imaging at the initial medical examination. The inclusion criteria were first-ever confirmed supratentorial stroke and a MMSE score of ≥ 24. The exclusion criteria were: a diagnosis of other central nervous system disorders, such as traumatic brain injury, hypoxic brain injury, or brain neoplasm; a history of neurodegenerative disorders; the presence of bilateral hemispheres or infratentorial lesions; bodily or visuospatial hemineglect and apraxia; and a history of injury, major trauma, or surgery in the upper limb on the paralyzed side.

The study protocol was approved by the research ethics committees of Kobe Gakuin University (approval number: 20-29) and Kishiwada Rehabilitation Hospital (approval number: 2021-002). Written informed consent was obtained from all patients. This study was conducted in compliance with the Declaration of Helsinki and its subsequent amendments.

### Group allocation

Post-stroke CRPS was diagnosed according to the Budapest Criteria of the International Association for the Study of Pain, 3 months after admission^28^. Patients exhibited persistent pain disproportionate to any inciting event, along with two or more signs in at least three of four categories: sensory, vasomotor, edema/swelling, and motor/trophic. These signs included changes in skin color, temperature, texture, swelling, decreased range of motion, and abnormal hair or nail growth. Furthermore, there was no other diagnosis that better explained the patient’s signs and symptoms. These patients were assigned to the post-stroke CRPS group. Patients with no sign or only one sign were assigned to the non-post-stroke CRPS group.

### Demographic and clinical data

All patients were assessed for demographic data (age, sex, etiology of stroke, side of the lesion, days since onset, and MMSE) and three outcome measures of upper limb disuse that reflect disuse (FMA-UE, ARAT, and MAL), lower limb disuse (FMA-LE), balance performance (BBS), and physical inactivity (IPAQ-SF). The NRS used to determine pain intensity was assessed only in patients with post-stroke CRPS. NRS is a tool for obtaining the subjective response of the degree of pain on a scale from 0 (no pain at all) to 10 (unbearable pain). All the abovementioned assessments were conducted at the time of admission.

### Measurement of predictor variables

The 33-item FMA-UE (3-point ordinal scale; score range: 0–66 points) and the 17-item FMA-LE (2-point ordinal scale; score range, 0–34 points) have been widely used to assess the severity of upper or lower limb paralysis, respectively^29,30^. A higher score indicated a lower level of impairment in the paralyzed upper or lower limb. The 19-item ARAT (4-level ordinal scale; score range: 0–57 points), was used to assess motor function in the paralyzed upper limb. The ARAT is a performance test representative of the major activities of the upper extremities in activities of daily living (ADL)^31^. A higher ARAT score indicates a lower level of impairment in the paralyzed upper limb. MAL was used to assess the frequency of paralyzed arm and hand use in daily life; it is a structured interview that elicits information regarding approximately 14 ADL^32^. Patients were instructed to assess how well (QOM) and how much (AOU) they used their affected upper limb to perform each ADL. A higher MAL score indicates more frequent use of the paralyzed arm and hand. The 14-item BBS (4-point ordinal scale; score range, 0–56 points) was used to assess static balance during sitting and standing, as well as dynamic balance during transitions and standing^33^. The BBS is a valid measure of balance in stroke and has high intra- and inter-rater reliability as well as excellent sensitivity to change^34^. The IPAQ is the most widely used physical activity questionnaire^35^, with two versions available: the long form comprising 31 items (IPAQ-LF) and the short form comprising nine items (IPAQ-SF). The IPAQ-SF was used in this study. The IPAQ-SF records the activity times for four intensity levels: (1) vigorous-intensity activity, such as aerobics; (2) moderate-intensity activity, such as leisure cycling; (3) walking; and (4) sitting and lying positions. In this study, only the sitting and lying positions were evaluated to assess the degree of physical inactivity.

### Statistical analyses

Data are presented as the mean ± standard deviation. The Mann–Whitney U test or t-test was used to compare continuous variables in the post-stroke CRPS and non-post-stroke CRPS groups based on the Shapiro–Wilk test. Fisher’s exact test was used to compare categorical variables. The level of statistical significance was set at p < 0.05.

A decision tree algorithm was used to illustrate the relationships among factors contributing to the development of post-stroke CRPS and their hierarchical classification. The CART method is a binary partitioning statistical technique that begins with the entire sample and progressively divides it into sub-samples that are homogeneous with respect to a predefined outcome. It identifies the variables that have the greatest impact on the forecast. The input variable that facilitates the most effective division is dichotomized through automated analysis at an optimal threshold. We conducted a CART analysis to develop a model for the classification of post-stroke CRPS and non-post-stroke CRPS 3 months after admission using IBM SPSS Statistics software version 25 (IBM Corp., Armonk, NY, USA). CART analysis, which uses a tree-like model, is a statistical method used to identify an explanatory variable that affects the objective variable. Variables that showed significant differences in the demographic and clinical data at the time of admission between the groups were treated as explanatory variables. Ten-fold cross-validation of the decision tree was performed to confirm the misclassification risk of the CART model estimated for the entire sample and to cope with the overfitting and instability inherent to the decision tree.

## Data Availability

The datasets generated and/or analyzed during the current study are available from the corresponding author upon reasonable request.
